# The “Dental Register” and Quinine

**Published:** 1905-04

**Authors:** 


### Editorial.

### THE "DENTAL REGISTER" AND QUININE.

In the *Dental Register* for January, 1905, there is an editorial of which the following is an abstract. The editor says, as a portion of his criticism, that—

> Dr. Joseph Head, in the December *Items*, makes the following statement: after thoroughly cleansing the roots of teeth affected with pyorrhœa, firmly fix the teeth with ligatures or splints and pack the pockets full with quinine sulphate as an antiseptic dressing. The quinine will exclude bacteria and cause the gums to become reattached to the peridental membrane or tooth substance. This is a rather strong statement and one that the pathology of this disease and the pharmacology of the drug will scarcely justify on logical grounds; but Dr. Head quotes Dr. James Truman as the author of the remedy and certifies it from his own clinical experience. We sometimes meet with unexpected therapeutic paradoxes in the clinical use of drugs, which lead us to lose confidence in scientific findings. . . . Quinine may be an ideal remedy for pyorrhœa; but how many patients will submit to such a treatment! The taste of this drug is exceedingly distasteful to almost every one, even when taken in regular form and dosage for systemic impressions. We do not think many patients will be willing to have their gums impregnated with this bitter drug to be gradually dissolved in the mouth fluids and so manifest its bitterness for several hours or perhaps days. The remedy will be worse than the disease, and the dentist who makes use of it ought to be very sure of the loyalty of his patient. Dentists have not as yet reached the sugar-coated stage in therapeutics, and it is possible that their peculiar field is not adapted to that system; but there is too much of the old style use of radical remedies, or it may be thoughtless use of powerful or distasteful drugs."

When the foregoing met the writer's eye, as he was scanning the pages of our esteemed contemporary, it was passed over with many other not over-well-digested paragraphs running through dental literature, but since then this view of the editor of the *Dental Register* has been extensively copied, and with evident approbation, and, to that extent, a loss of "confidence in scientific findings," as the editor expresses it rather sarcastically. This forces the writer to enter a defence of this drug and to meet the implied charge of empiricism contained in the editorial quoted.

The article, in question, is so manifestly the result of prejudice and ignorance as to the therapeutic value of quinine, and all the derivatives of cinchona, that the writer will not attempt to discuss the statements made. They must be left for good or ill, as the reader may choose to interpret them.

It is, however, important that the reasons should be given for the use of this drug, that the sceptical may, at least, be made aware that its therapeutic value in the disease mentioned has been very thoroughly tested, and that for twenty-five years, and yet the writer of this has not reached "the sugar-coated stage" in its use in the treatment of patients.

If our friends of the medical profession were to confine their therapeutics to pleasant-tasted drugs, the methods of treatment would be forced to undergo an entire change. The business of the conscientious practitioner is to endeavor to bring about a restoration to normal conditions, and he is not required to consult the æsthetic tastes of his patients in its accomplishment.

The treatment of pyorrhœa alveolaris is an interesting theme, even though hackneyed, for each individual attempting restorations to health is apparently faithless as to the value of any other method save that which he originated, and he is not, as a rule, quite satisfied with his procedures whether original or adopted. Thus in reviewing the literature of the subject, one is forced to the conclusion that the dental world must still wait expectantly for the man who will be able to satisfy criticism and produce a process of treatment satisfactory to practitioner, teacher, and student. The writer would, therefore, prefer to keep silence and let facts speak for themselves, but he cannot afford to permit a lack of knowledge as to the real therapeutic value of quinine to still further increase present prejudice.

No one, certainly not the writer, has ever claimed quinine as a specific in the disease named. It has been recommended for certain properties believed to be of peculiar value and not common to other drugs. What are these?

1. It acts on broken tissue more kindly than any other antiseptic. It further acts (Binz) to prevent diapedesis of the leucocytes of the blood, and thus, by inhibiting their amœbic action, pus is not, as a rule, produced in the pyorrhœal pockets.

2. It acts as a powerful antiseptic, and will prevent the ingress of pathogenic organism for at least twenty-four hours, thus giving time for healthy granulations and the eventual filling up of the pockets of normal tissue. It is at the same time absolutely non-irritating.

No claim has ever been made that this agent will alone cure pyorrhœa alveolaris. It is simply one part of the treatment, but by no means the least active in the process of restoration.

The writer has used quinine and the derivatives of cinchona for the reduction of inflammatory conditions of the oral cavity since 1882, and in 1884 called the attention of the American Dental Association to the value of these in the therapeutics of dentistry. For years quinine sulphate has taken a place in the treatment of pyorrhœa alveolaris in the Department of Dentistry, University of Pennsylvania, almost to the exclusion of other drugs for a similar purpose. The writer has tried many old and new drugs as substitutes, including mercurial preparations, but in no one have the results justified hopes and expectations.

During these years there have been treated in this dispensary from fifty to one hundred cases of pyorrhœa during each session, by the method adopted and advocated by the writer, and, as all the pathological cases, of whatever kind, treated by the Senior Class are required to be registered and written out, there is compiled a series of valuable statistics, that fully warrant the assertion that no more thorough clinical experimentation has ever been made of any disease coming under the care of the average practitioner. All cases are not cured, nor will it be possible to accomplish this by any method of local or systemic treatment, for the disease assumes several forms from the simple pericemental inflammation to the senile form (osteomalacia, Talbot). The operator must be able to make a careful differential diagnosis before he assumes the responsibility of treatment. The percentage of success in the clinical work of the department aforesaid has been, in pyorrhœa alveolaris, fully ninety-five per cent., a result quite satisfactory, and, it is thought, not exceeded by other methods of treatment.

The objections to the taste of the drug have no force. It is true, very few patients find it agreeable, but some do, and they all acknowledge it gives them comfort and return for renewal with satisfaction over the *bitter* experience.

The writer has made it the rule of his life not to defend, by lengthy and oft-repeated essays, any proposition which had been originated by him. He has felt satisfied to give it publicity, and there let it rest, confident that sooner or later the truth would find its place. It took twenty years for his views on bleaching teeth to take root as a method of practice in the dental profession, and when the light began to dawn, among the first to discover it was a practitioner in the Middle West, who so fully appreciated the ideas contained in the writer's article in the *American System of Dentistry,* that he took it bodily, placed his name at the top, and read it before one of the societies of that region, and it was published to go down to future generations as the product of John Doe, bleacher. It has taken another twenty years to reach an editorial opposing the use of quinine. There is cause for encouragement.

To satisfy our contemporary, it is hoped, or, in any event, to hasten his enlightenment in this direction, there follows some of the opinions of eminent scientific authorities upon which the use of quinine is based. If, after giving due consideration to these, it is hoped that the editor may conclude that if the so-called "Truman Process" be worthless, it at least has the merit of being founded on eminent authority, and cannot by any twisting of words be classed as a method of practice "worse than the disease."

"It has recently been experimentally proved by Binz, Hallier, Pavesi, and others that one part of the alkaloid (quinine sulphate) in three hundred parts of milk, albuminous solutions, meat, honey, syrup, etc., will keep in check for a long time putrefaction and other fermentations. Professor Binz has shown that this is due to a poisonous influence upon the low forms of life." (United States Dispensatory.)

"Quinia is supposed to control inflammation by its destructive influence on movements of the white corpuscles, and Binz maintains that, after irritating and inflaming the mesentery by the administration of quinia, the white corpuscles are killed and their migration by the tissues is prevented. It is supposed to lower temperature by lessening the ozonizing power of the blood, and thus checking oxidation." (Ringer, "Hand Book of Therapeutics," page 583.)

"As Binz originally noted, quinine when added to freshly drawn blood arrests the amœboid movement of the white cells. Moreover, when applied in very dilute solution to the exposed mesentery of a frog, it suspends, almost immediately, the migration of the leucocytes. This cessation of diapedesis was believed by Binz to be due to poisoning of the corpuscles, but Metschnikoff and his followers attribute it rather to the repelling influence (negative chemotaxis) which quinine, in common with many other substances, exerts upon motile cells." ("Modern Materia Medica and Therapeutics," Stevens.)

"In the blood, quinine arrests the migration of the white corpuscles and checks its amœboid movements; the red cells are rendered less adhesive, and their oxygen-carrying function is impaired. The experiments of Lokoloff upon rabbits show that quinine exerts a favorable influence upon the healing of wounds. Inflammatory degeneration of tissue is notably decreased." ("Materia Medica and Therapeutics," Shoemaker.)

"Binz and his pupils have shown that quinia inhibits or lessens the activity of the white blood-corpuscles, and, indeed, destroys them, or arrests their production; for in cats poisoned by this agent the number of white corpuscles was found to be considerably less than in unpoisoned animals (Scharrenbroich, Martin, Jerusalimsky, Geltowsky). By all the observers just named, by Baxter, who made a series of carefully conducted experiments, and by Cutter, it has been established that quinia inhibits the amœboid movements of the white corpuscles. . . . Quinia has also the power to prevent or arrest the migration of the white corpuscles from the vessels." ("Materia Medica and Therapeutics," Bartholow.)

"The correctness of the original observations of Professor Binz upon the out-wandering of the white blood-corpuscles in the Cohnheim frog must be considered as established, but it is not proved that the failure of the blood-corpuscles to escape from the irritated vessels is due to the arrest of their amœboid movements by the quinine. In a series of experiments Dr. H. A. Hare found that the vessels in the cinchonized frog were much more contracted and had their walls much thicker than in a corresponding frog without quinine. The contraction of the vessels is thought by Dr. Hare to be the result of a direct action exerted by the drug upon the muscular coat of the arterioles. It is certain the alkaloid reduces very markedly the force of the heart. It is, therefore, possible that the quinine prevents the out-wandering by lessening the force which is driving the corpuscles and at the same time increasing the resistance of the capillary walls." ("Therapeutics: Its Principles and Practice," H. C. Wood.)

It would seem unnecessary to multiply authorities further. It must be clearly evident that the consensus of opinion is all in favor of the conclusions of Binz, and it is upon these investigations that the use of the derivatives of cinchona is based. Upon broken tissue quinine acts decidedly.

Before leaving the subject, it may be well to state exactly the place quinine occupies in the treatment of pyorrhœa alveolaris, as understood and practised by the writer. It may also enlighten our critical editor and disabuse his mind of "paradoxes."

Briefly, the treatment starts upon the theory that pyorrhœa alveolaris is based upon local irritation, possibly through calculus and always through pathogenic bacteria, and that this irritation causes active chemotactic influence upon the leucocytes of the blood and consequent free migration of these corpuscles. To meet these conditions the pockets are treated—

1. By free injection of hydrogen dioxide not over three per cent. in strength.

2. Cauterization by sulphuric acid, commercially pure, twenty-five, thirty, or fifty per cent. solution. Action continued from thirty to sixty seconds.

3. Neutralization of acid by a magma of sodium bicarbonate and water, packed in pockets.

4. After free boiling through this action the pockets are freely washed with warm water.

5. Quinine sulphate made in a paste with water, placed in every portion of the pockets and left there for two days.

6. To be followed by a good antiseptic mouth-wash for an indefinite period. The character of this is not so important, but the writer prefers an agent that will be positively inhibiting to pathogenic micro-organisms, beta-naphthol (hydronaphthol) preferred.

This article is not an essay on pyorrhœa alveolaris, hence manipulative details are omitted. The writer is not at all anxious that any one, including our worthy editor, should be convinced of the value of this method of treatment, but he is concerned in an effort to have a much abused agent find a place in the therapeutics of dentistry and be a source of comfort to many suffering patients.

In this connection it may be well to state that McKesson and

Robbins, New York City, have prepared, at the suggestion of Dr. Joseph Head, small delicate wedges of quinine. These are very convenient, and are more readily applied than in the paste form, and should be equally as satisfactory in results.

